# Antibiotic resistance and tolerance: What can drug delivery do against this global threat?

**DOI:** 10.1007/s13346-023-01513-6

**Published:** 2024-02-10

**Authors:** Juan Aparicio-Blanco, Nikhar Vishwakarma, Claus-Michael Lehr, Clive A. Prestidge, Nicky Thomas, Richard J. Roberts, Chelsea R. Thorn, Ana Melero

**Affiliations:** 1 Department of Pharmaceutics and Food Technology, Faculty of Pharmacy, Complutense University of Madrid, 28040 Madrid, Spain; 2 Department of Pharmacy, Gyan Ganga Institute of Technology and Sciences, Jabalpur, 482003 Madhya Pradesh India; 3 Department Drug Delivery across Biological Barriers (DDEL), Helmholtz Institute for Pharmaceutical Research Saarland (HIPS), Saarbrücken, Germany; 4 Department of Pharmacy, Saarland University, Campus Building E8 1, 66123 Saarbrücken, Germany; 5 Centre for Pharmaceutical Innovation, Clinical and Health Sciences, University of South Australia, Adelaide, SA 5000 Australia; 6 New England Biolab, 240 County Road, Ipswich, MA 01938-2723 USA; 7 Biotherapeutics Pharmaceutical Research and Development, Pfizer, Inc., 1 Burtt Road, Andover, MA 01810 USA; 8 Department of Pharmacy and Pharmaceutical Technology and Parasitology, Faculty of Pharmacy, University of Valencia, 46100 Burjassot, Spain

**Keywords:** Antibiotic resistance and tolerance, Drug delivery, Biofilm, Bacterial cell membrane, Bacterial envelope, Nanomedicine, Bacteriophages

## Abstract

Antimicrobial resistance and tolerance (AMR&T) are urgent global health concerns, with alarmingly increasing numbers of antimicrobial drugs failing and a corresponding rise in related deaths. Several reasons for this situation can be cited, such as the misuse of traditional antibiotics, the massive use of sanitizing measures, and the overuse of antibiotics in agriculture, fisheries, and cattle. AMR&T management requires a multifaceted approach involving various strategies at different levels, such as increasing the patient’s awareness of the situation and measures to reduce new resistances, reduction of current misuse or abuse, and improvement of selectivity of treatments. Also, the identification of new antibiotics, including small molecules and more complex approaches, is a key factor. Among these, novel DNA- or RNA-based approaches, the use of phages, or CRISPR technologies are some potent strategies under development. In this perspective article, emerging and experienced leaders in drug delivery discuss the most important biological barriers for drugs to reach infectious bacteria (bacterial bioavailability). They explore how overcoming these barriers is crucial for producing the desired effects and discuss the ways in which drug delivery systems can facilitate this process.

**Graphical abstract:**

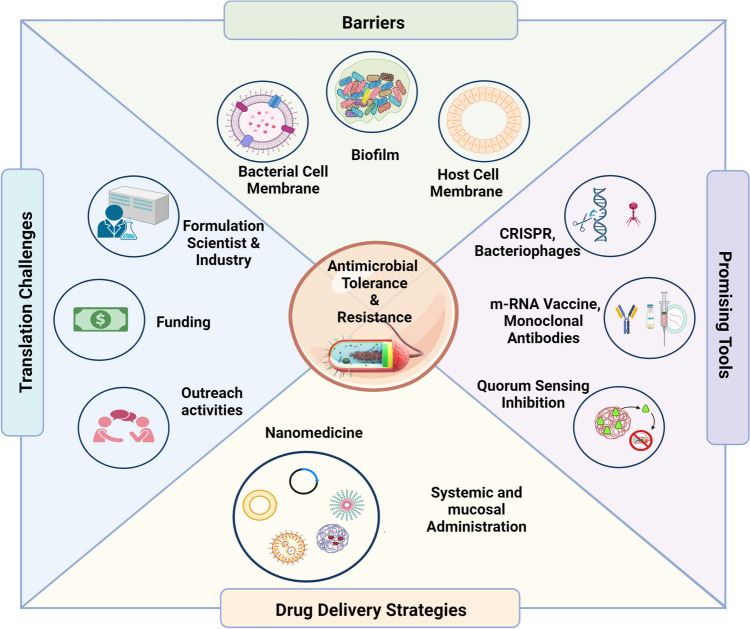

## Introduction

Antimicrobial resistance and tolerance (AMR&T) occur when microorganisms, such as bacteria, viruses, fungi, or parasites, develop mechanisms to evade the effects of drugs used to treat the infections they cause. Tolerance is an innate phenomenon to the microbe, while resistance is developed [1]. The continuous use of antimicrobials for the management of infectious diseases has led to the development of AMR&T. In addition, the mutation and spontaneous evolution of the bacteria and gene transfer also play a significant role [2]. As a result, AMR&T has emerged as a growing health concern globally. AMR&T has severely impacted the treatability of infectious diseases since the misuse and unregulated overuse of antimicrobials in agriculture, animals, and humans have led to dire consequences, rendering existing antimicrobials ineffective. Other consequences of the development of AMR&T include a significant increase in morbidity and mortality due to treatment failures, limited availability of adequate treatment options, increasing healthcare costs, overwhelmed healthcare systems, and a threat to animal, agricultural, and food safety [3]. AMR&T accounted for approximately 4.96 million deaths globally in the year 2019 and this number is projected to rise to 10 million by the end of year 2050 [4, 5]. To address the global challenge of AMR&T, the World Health Organization in 2015 developed a global action plan (GAP-AMR) and launched the “Global Antimicrobial Resistance and Use Surveillance System” (GLASS) on October 22, 2015. GLASS is a collaborative effort and serves as a cornerstone for evaluating the spread of AMR and monitoring the effectiveness of strategies through surveillance at local, national, and global levels [6]. On September 21, 2016, the United Nations General Assembly convened to discuss the problem of antibiotic resistance and deemed it “the greatest and most urgent global risk” [7].

The development of novel antimicrobial drugs is lagging behind the emergence of AMR&T, particularly among major bacterial pathogens; therefore, the main focus of this perspective article is on antibacterial agents. In recent times, various antibacterial resistance profiles have been identified, encompassing multidrug-resistant, extensively drug-resistant, and pan drug-resistant phenotypes [8]. The typical targets of antibiotics include bacterial ribosomes (aminoglycosides), cell wall synthesis (β-lactams), lipid membrane structure (quaternary ammoniums), DNA replication (quinolones), and the single-carbon metabolic pathway (sulfonamides) [9]. Consequently, bacteria have developed various inherent strategies to resist antibiotics in response to those targeted attacks. They employ aggressive resistance mechanisms by producing enzymes that degrade antibiotics or modify their structure, particularly in the case of β-lactams. In addition, bacteria can adopt passive or protective resistance mechanisms, including target mutations, activation of efflux pumps, and reduction of membrane permeability. Moreover, these microbes can mobilize multiple resistance mechanisms simultaneously against each antibiotic [10].

The limitation of current clinical options for confronting resistant infections has led to a critical problem that should raise researchers’ interest in working on new approaches to face the growing problem of bacterial resistances [11]. Overcoming AMR&T requires a multifaceted approach involving various strategies and interventions. Key strategies include enhanced surveillance, the implementation of programs to optimize the use of antibiotics and prevent their misuse or overuse, the development of new antimicrobial agents to combat drug-resistant infections, reducing the overall burden of infectious diseases through vaccination, increasing public awareness and education, and fostering international cooperation [12]. It is important to note that overcoming AMR&T is a complex and ongoing challenge. Continued investment in research, surveillance, and the implementation of comprehensive strategies is necessary to mitigate the impact of drug-resistant infections and safeguard public health [13]. Thus, a collaborative approach including policymakers, regulatory agencies, healthcare professionals, researchers, the pharmaceutical industry, and society is required to alleviate the severity of AMR&T and protect antimicrobials’ effectiveness for future generations.

Novel advanced drug delivery strategies are urgently needed to enhance antimicrobial efficacy, combat developed AMR&T, and minimize the further development of drug resistance. Drug delivery scientists can certainly play a key role in the fight against AMR&T and future prevention through the design of novel advanced drug delivery systems employing both classical and new anti-infectives. In this regard, the perspectives of globally leading researchers concerning the key upcoming challenges of AMR&T, as well as current and future promising strategies on how to overcome them, are shared herein. Significant emphasis is placed on the role of translational drug delivery technologies in advancing the development of promising therapeutic tools and prophylactic measures, including RNA/DNA therapies or bacteriophages.

To discuss the current scenario of AMR&T and the strategies needed to overcome such an enormous challenge, this article includes perspectives from Professor Claus-Michael Lehr, Professor Clive Prestidge, Dr. Nicky Thomas, and Professor Richard J. Roberts—world-leading experts in the field of anti-infective drug delivery.

Claus-Michael Lehr is a Professor at Saarland University as well as co-founder and head of the department “Drug Delivery” at the Helmholtz Institute for Pharmaceutical Research Saarland (HIPS), which was established as a branch of the Helmholtz Centre for Infection Research (HZI) Braunschweig in 2009. Lehr has also been a co-founder of Across Barriers GmbH and PharmBioTec GmbH to provide dedicated contract research services to the industry. Lehr’s department focused on the delivery across biological barriers since he became a Professor at Saarland University in 1995, and then deepened into infectious diseases after founding the Helmholtz Institute of Pharmaceutical Research Saarland (HIPS) in 2009. There, he started investigating particularly the biological barriers that need to be overcome for antimicrobial delivery. His group has introduced the concept of bacterial bioavailability [14] to refer to the ability of drugs to reach the bacterial targets.

Clive Prestidge is a Professor of Pharmaceutical Science within Clinical and Health Sciences, co-director at the Centre of Pharmaceutical Innovation at the University of South Australia (UniSA), head of the Nanostructure and Drug Delivery research group, and the founder of Ceridia Pty Ltd., a clinical stage biopharmaceutical company established to commercialize the Lipoceramic drug delivery technology he invented. The goal of Prof Prestidge’s laboratory is to (1) deliver challenging therapeutic molecules for better medicines, (2) eradicate bacterial biofilms, (3) advance nanomedicines, and (4) optimize biotech and pharmaceutical technologies.

Nicky Thomas is an Adjunct Senior Research Fellow at UniSA’s Clinical and Health Sciences and previously held positions across UniSA and The Basil Hetzel Institute for Translational Health Research. His research is concerned with the interaction of nanomedicines with bacterial biofilms and with the development of novel strategies to combat some of the most debilitating diseases—chronic infections. His second research interest is concerned with the question of how nanomedicines can be used to improve the efficacy and safety of drugs with pharmaceutically challenging properties.

Sir Richard Roberts is Chief Scientific Officer at New England Biolabs. He is a member of the American Academy of Arts and Sciences, The Royal Society, and The National Academy of Sciences and won the Nobel Prize in Physiology or Medicine (1993) for the discovery of introns in eukaryotic DNA and the mechanism of gene splicing, which has had a profound impact on the study and applications of molecular biology.

## Intrinsic biological barriers for targeting bacteria, shortcomings of current drug delivery strategies, and challenges ahead

Given the well-described biological complexity of AMR&T, all authors have emphasized the utmost importance of overcoming biological barriers to ultimately deliver anti-infectives to their targets. According to Roberts, the first step is to identify the pathogen causing the infection so that treatment can be specifically selected. Lehr and Prestidge have also emphasized that the proper identification of infection etiology will help determine the biological barriers to overcome on a case-by-case basis. In this context, Thomas has highlighted that as our therapies improve, so should the diagnostic tools used to determine the type of pathogens causing the infection.

According to Lehr, in the context of bacterial infectious diseases, in addition to the general biological barriers for achieving targeted delivery of the drugs to the infection site, there are clearly up to three distinct biological barriers that need to be investigated, and for which drug delivery strategies can be developed. Lehr coined the term “bacterial bioavailability” [14] in this regard and agreed with Prestidge and Thomas that drug delivery systems are key to help overcome some of these biological barriers and, ultimately, AMR&T. Accordingly, they are all confident that drug delivery scientists will play a major role in this major challenge.

The first biological barrier refers to bacterial biofilms, a growth state where bacteria exist in communities to secrete an extracellular polymeric substance matrix made of sugars like agarose or lectins. This matrix ultimately constitutes a hydrogel with diffusional barrier properties analogous to mucus. As a result, biofilms significantly limit bacterial bioavailability of anti-infectives, leading to high tolerance and increasing the required level of antimicrobial agents to overcome a biofilm infection by 100- to 1000-fold compared to the levels needed to overcome that infection in planktonic form (independent bacteria). Therefore, the delivery of antimicrobials to the biofilm location must be maximized. Indeed, Thomas pointed out that in some conditions, such as cystic fibrosis lung infections, bacterial biofilms have now been clearly established as a major source of recurring infections [15, 16]. For this first biological barrier, Lehr suggests adopting many of the delivery strategies already explored for mucosal delivery.

The second barrier refers to the bacterial cellular envelope, also termed the bacterial cell wall, especially in the case of Gram-negative bacteria, given that they supplement their peptidoglycan cell membrane with an outer membrane containing lipopolysaccharide [17]. Increasing diffusion across the bacterial cellular envelope allows reaching intracellular bacterial targets, thereby enhancing bacterial bioavailability. In the development of new antimicrobials, Professor Lehr touches on the idea of changing bacterial behavior, instead of using current strategies that lead to AMR&T.

**Fig. 1:**
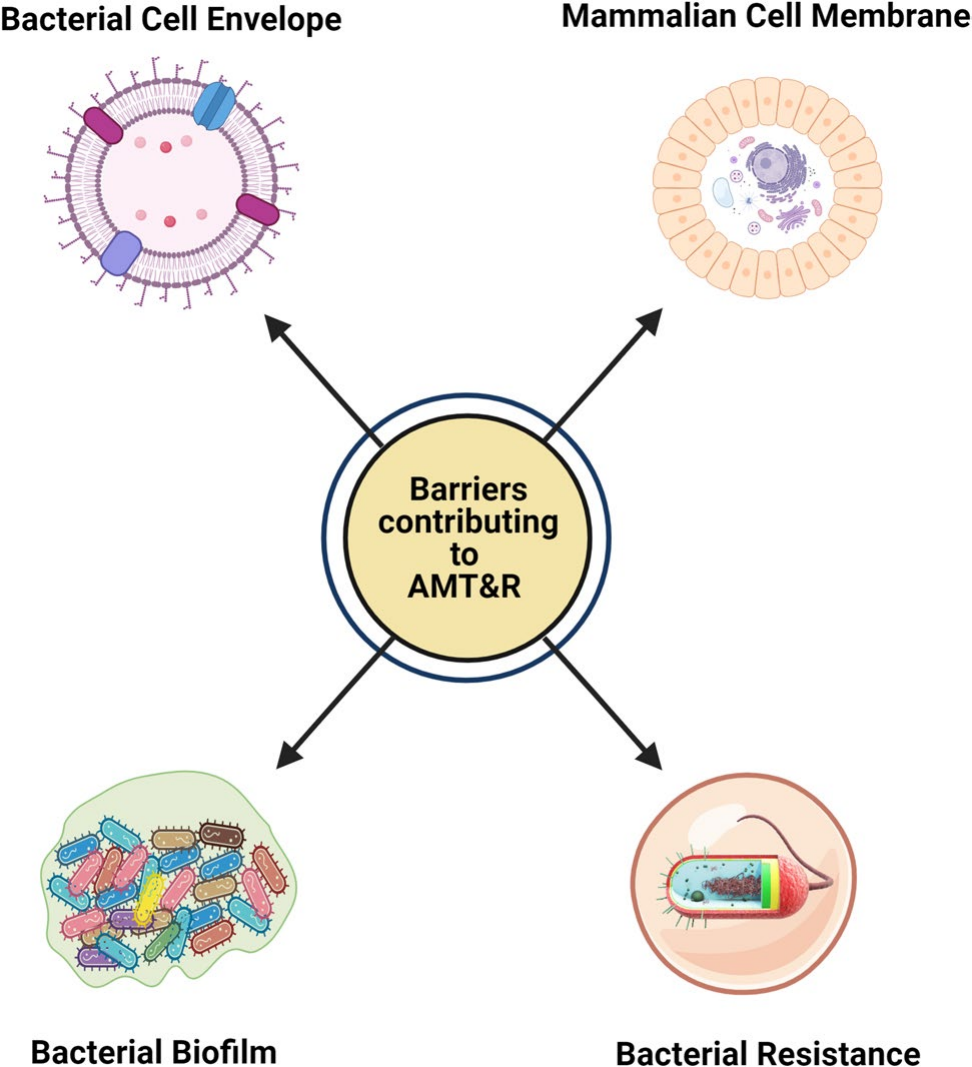
Different barriers for AMR&T

Thirdly, there is another barrier provided by the host cell membrane to pathogens residing intracellularly, protecting themselves from the innate immune system. Prominent examples of intracellular infections are those associated with Mycobacteria, where the pathogen can “camouflage” itself inside human immune cells, evading the effects of the immune system. These types of infection may remain dormant and undiagnosed but drive subsequent infections. In these cases, not only the identification of the pathogens but also the ability of antimicrobials to diffuse across human cells is of utmost importance (Fig. 1) [18–20].

Regarding the shortcomings of current drug delivery strategies, Prestidge has called on us all to analyze how antimicrobials are currently dosed. They generally consist of systemic (either oral or intravenous) administration of antimicrobials, devoid of any drug delivery technology. Hence, the delivery relies solely on the intrinsic biodistribution of these anti-infectives to reach their targets [21]. Altogether, this approach certainly limits the amount of drug that reaches the site of infection, leaving a significant opportunity for drug delivery scientists to consider more localized and targeted delivery of antimicrobials to the site of action. For example, in the context of topical or lung infections, higher local levels of anti-infectives, together with reduced systemic exposure, are expected to be achieved when delivered topically or via inhalation, respectively, as the simplest form of drug targeting. This has been a main theme in Dr. Thomas and Prof. Prestidge’s lab [22–26]. Indeed, all authors agreed that we are not currently maximizing the efficacy of our antimicrobials and pointed to AMR&T being the result of a partial failure in a delivery situation since exposing microorganisms to subtherapeutic levels is a breeding ground for resistance. Taken together, Prestidge stated that there is clearly potential for drug delivery systems in the field of classical antimicrobials, whereas in the case of novel therapies (i.e., monoclonal antibodies or antimicrobial peptides), drug delivery technologies are going to be critical in transitioning those from concepts into effective clinical use due to their additional inherent instability in biological fluids. To overcome the drug delivery challenges ahead, Thomas pointed out that pharmaceutical scientists should work in liaison with pathologists and microbiologists to properly understand these targets, as there is no chance for these barriers to be overcome without a proper understanding of them.

As a first approach to improving current anti-infective therapies, Lehr pointed out the utilization of existing drugs that are potentially active but suffer from adverse effects or poor bioavailability, limiting their clinical use as therapeutics, such as the ototoxicity of aminoglycosides such as gentamicin. Obviously, some parallels can be drawn to drug delivery in cancer contexts, where the challenges of resistance and toxicity are analogous to those in antimicrobials. Targeted delivery to the site of infection might improve therapeutic efficacy, as we may learn from strategies previously described in cancer therapies [27]. This enhancement in bioavailability at the site of infection is desired to significantly widen the therapeutic window of anti-infectives.

When delivering antimicrobial nanocarriers, it is also essential to consider the influence of the protein corona, which plays a critical role in controlling the biodistribution of antibiotics; this is particularly important for IV delivery and delivery to the lung [28].

## Promising therapeutic tools to fight against resistant bacteria

With modern medicine, novel tools and concepts are emerging that could help overcome antimicrobial-resistant bacterial infections.

Lehr is very excited about the CRISPR (clustered regularly interspaced short palindromic repeats) concept. A Nobel Prize-winning phenomenon, CRISPR/Cas9, was originally discovered as a defense system of bacteria against phage viruses attacking the cell [29]. Prof Jennifer Doudna and Prof Emmanuelle Charpentier then demonstrated it was also possible to convert this similar concept to eukaryotic cells, thereby enabling gene editing [30]. There are other CRISPRs and other Cas proteins that can be activated, allowing researchers to find a way to destroy RNA in bacteria or viruses, which may enable the development of such a therapy [31]. The use of CRISPR technology is of interest to the drug delivery community because it requires unique nanomedicine delivery, including lipid nanoparticles to surpass the cell wall barrier, delivering molecular genetic tools into the cell. With the messenger RNA (mRNA)-based Covid-19 vaccine, we have succeeded in single nucleotide delivery [32, 33]. The next challenge, however, arises with multi-nucleotide delivery. Besides the mRNA encoding the CRISPR proteins, the guide RNA needs to be delivered, possibly using the same nanocarrier, but with different release kinetics. Another example for multi-nucleotide delivery might be in developing vaccines where, in addition to the antigen, some adjuvant proteins are administered. This can perhaps be realized by co-delivery of plasmid DNA and mRNA to address the challenges of different expression kinetics for the encoded proteins [34]. While stable chemistries and conjugates enable the modification and delivery of small oligonucleotide RNA therapeutics such as antisense oligonucleotides, siRNAs, and endogenous adenosine deaminases acting on RNA-oligonucleotides, mRNA-based and DNA-based therapeutics necessitate a delivery vehicle to enter cells. To streamline this entry process, researchers have devised various RNA delivery systems employing diverse materials, including polymers and lipid nanoparticles. Two RNA drug delivery systems have been approved by the FDA to fight against pathogens: the COVID vaccines from Moderna and Pfizer-BioNTech [35].

Roberts suggested as an alternative approach to get anti-infectives into bacteria the inherent tropism of bacteriophages (or phages), namely, the viruses that specifically target and infect bacteria, but not eukaryotic cells [36]. These are naturally occurring bacterial parasites that cannot replicate on their own and are dependent on a bacterial host for their survival. As a result, they can selectively infect, replicate, and kill bacterial but not human cells, which may help prevent side effects. Phage therapy is the therapeutic use of naturally occurring bacteriophages for the treatment of bacterial infections. As bacteriophages are self-replicating entities, they produce a natural amplification of the therapeutic effect [37, 38]. Recent advancements in biotechnology have broadened the range of potential phage therapeutics, including innovative approaches involving bioengineered phages and purified phage lytic proteins [39]. This type of therapy has been in practice for nearly a century. However, the widespread decrease in the effectiveness of antibiotics has sparked a renewed interest in re-evaluating and exploring its potential. Furthermore, innovations in the gene-editing tool CRISPR/Cas have created novel opportunities for phage therapy. One example of which is the use of bioengineered phage to deliver a CRISPR/Cas programmed to disrupt antibiotic resistance genes and destroy antibiotic resistance plasmids [40].

Prestidge is further interested in how intensive the research is becoming in monoclonal antibodies, antimicrobial peptides, antimicrobial polymers, and novel biologics that target unique bacterial tolerance mechanisms (i.e. biofilm-dispersing enzymes). Several monoclonal-antibody-based biologics are FDA approved, such as raxibacumab or obiltoxaximab, approved for *Bacillus anthracis* and bezlotoxumab (Zinplava^®^) for *C. difficile* toxin B. Others are in phase III clinical trials for the treatment of sepsis [41], including suvratoxumab (MEDI4893) or tosatoxumab (Salvecin, AR-301), which target *Staphylococcus aureus* alpha toxin.. According to Prestidge, the technology development of the company Kane Biotech in this area should be followed with special interest. Their DispersinB^®^ Hydrogel to treat biofilm-mediated antimicrobial resistance in non-healing chronic wounds has received the Medical Technology Enterprise Consortium Research Project Award, granted in 2020 and funded by the US Department of Defense [42].

Antimicrobial polymers are substances capable of preventing or eliminating bacteria, which can manifest antibacterial properties through their intrinsic chemical structure, such as quaternary nitrogen groups, halamines, and polylysine. Alternatively, they can function as a framework to enhance the effectiveness of conventional antibiotics [43]. The translation of antimicrobial polymers from laboratory research to clinical application is still pending. Before progressing to clinical trials, research must still be conducted to assess in vitro and in vivo toxicity, biocompatibility, cell viability, biodistribution, and immunogenicity. Prestidge highlights from these therapeutic agents that they may be less likely to become resistant, but there are specific challenges around them, particularly their efficacy versus toxicity, and around delivering those to the point of action. In fact, these agents are notoriously difficult to deliver and are generally unstable in biological fluids. Therefore, their encapsulation in delivery systems will be very attractive to protect them against proteases or other challenges that could break them down before they reach the site of action. Additionally, some of these agents are positively charged, so they often stick in various body regions resulting in poor inherent biodistribution. Using a nanocarrier system can potentially overcome some of these challenges. This is one of the main research areas of the group led by Prestidge and Thomas. In this regard, their lab has collaborated with Prof Lynne Howell from The Hospital for Sick Children in Toronto, CA, to deliver biofilm-dispersing enzymes along with antibiotics—a combination treatment aimed at destroying bacterial biofilms and enabling antibiotics to be effective again [44, 45].

Lehr further points out the concept of so-called pathoblockers as an alternative to antibiotics. The aim here is to “disarm” rather than to kill the bacteria, thus exerting less pressure that might provoke AMR&T. A target for this strategy, jointly pursued by several groups at HIPS, is the so-called quorum sensing, a bacterial communication system that is essential for the formation of biofilms. The combination of some novel quorum sensing inhibitors with the “classic” antibiotic tobramycin not only reduced but completely eradicated *P. aeruginosa* biofilms. Co-delivery of the two drugs by innovative self-assembling nanocarriers, capable of penetrating the biofilm polymers, allowed for a reduction in the concentration of the antibiotics by about 50 times [46]. This example nicely demonstrates the potential of complex (nano)formulations in the context of AMR&T.

Thomas raises the point that the most promising tool is likely to be a combination of all these innovations. He believes the traditional broad-spectrum approach is not going to be useful in the future. Combining, for example, antimicrobial peptides and bacteriophages, or immunotherapy for a specific effect, may be required for effective treatment.

The emerging promising therapies in the antimicrobial space, including vaccines, CRISPR/Cas9, quorum sensing inhibitors, antimicrobial peptides, and phages, all require effective drug delivery to the site of action.

## Delivery strategies for anti-infectives in the pipeline

The development of innovative drug delivery strategies is essential for addressing the growing global concern of antibiotic-resistant bacteria and improving the effectiveness of antimicrobial therapies. Delivery systems aim to enhance the efficacy of antibiotics and other antimicrobial agents by improving their targeted delivery to specific infection sites, minimizing side effects, and optimizing therapeutic outcomes. Targeted delivery strategies have the potential to widen the therapeutic window for drugs that were previously considered too toxic for systemic delivery. There is a pressing need for delivery strategies and systems that guarantee effective doses while minimizing adverse effects. Delivery systems are also essential for enabling emerging therapies, i.e., RNA-based therapies. Explorations into the use of nucleotides as anti-infectives, with the potential for high selectivity in targeting bacteria for killing or modification, represent promising new concepts [47, 48]. However, successful implementation requires effective strategies for delivering these nucleotides into bacterial cells. Examples of such delivery systems include nanoparticles, liposomes, micelles, and polymer-based carriers, which can enhance drug stability, prolong circulation time, and facilitate controlled release. In addition, delivery methods, involving synergistic drug combinations, hold the potential to extend the utility of existing antibiotics—an important consideration given the challenges in discovering novel antibiotics. New delivery strategies have emerged, featuring highly tailored systems that utilize bacterial enzymes to trigger drug release, replicate pathogen entry pathways, or disrupt biofilm growth modes. An extensive discussion on detailed technologies for the treatment of AMR&T can be found in Loretz et al. [48].

Lehr made a plea for future directions in the field of drug delivery, emphasizing not only but especially antibiotics. These directions should focus not only on the cellular level but also on the final administration route that will be used to deliver the therapeutic agent. At the cellular level, the system needs to overcome some of the mentioned biological barriers (i.e., host or bacterial membranes, biofilms). However, the formulation scientist needs to guarantee that the delivery system reaches the cellular target using an adequate dosage form. For that purpose, drug delivery scientists should take advantage of advanced technologies to prepare efficient nanoscale drug carriers that can be further incorporated in traditional dosage forms. For instance, a delivery strategy recently explored for antibiotic delivery to combat *M. tuberculosis*, which resides in lung macrophages, was a nano-in-microparticle technology: whereas the nanoparticles mediated receptor-mediated uptake by phagocytosis after deposition in the lung, their incorporation into microparticles was necessary to render the necessary aerodynamic properties for pulmonary administration as inhalable aerosol [49].

Exploring future directions in drug delivery, intriguing concepts are emerging. For example, bioadhesive nanocarriers are being developed with lipids that originate from red blood cells, which show strong affinity and adhesion to bacteria [50]. These nanoscale systems have been designed with the aim of adhering to the surface of bacteria and ultimately acting as transenvelope systems (in an analogous manner as transdermal systems act) for bacteria. By accumulating the drug on the other side of the bacterial envelope, the delivery across this barrier is enhanced.

Phage therapy is also interesting for its specificity, as phages can identify and attach to particular bacterial strains through their surface receptors, rendering them highly specific [40]. In this regard, to enhance targeted delivery to the site of infection, we might also be able to learn from their strategies to optimize the design of drug delivery systems. Among the most promising advances in phage therapy is the isolation of phage-encoded lytic enzymes, which opens the possibility for the development of novel phage-based pharmaceuticals [51]. Moreover, bacteriophages can also act synergistically with classical antibiotics and have enzymes on their outer capsid that can degrade the extracellular polymeric substances present in bacterial biofilms [52]. However, it can be difficult to find an effective bacteriophage for a particular infection; a phage will kill a bacterium only if it matches the specific strain, which sometimes leads to the use of phage mixtures (“cocktails”) [53] to improve the chances of success and is often not welcome by regulatory agencies. Continuing obstacles encompass the necessity of expanding phage collections sourced from reference phage banks [54], the creation of phage screening techniques to identify therapeutic phages, and the assurance of stability throughout the manufacturing, storage, and transportation of phage preparations [55]. Phages are currently being used therapeutically to treat bacterial infections that do not respond to conventional antibiotics [56]. Several clinical studies have shown high inhibition of different antibiotic-resistant bacteria and minor side effects, which allows us to predict phage therapies as a potential replacement for antibiotic treatments [57–59]. Evaluating the side effects and potential impact of phages presents challenges, but thus far, phages have demonstrated a relatively low incidence of side effects. One possible explanation for this is the daily exposure of humans to phages, which suggests a lack of detectable adverse effects in humans. However, further research is needed to better understand their safety, efficacy, and optimal usage.

Formulation scientists can draw inspiration from phage therapy to develop biomimetic nanoparticles, thus eliminating the need for actual phages. By leveraging synthetic systems, scientists can design biomimetic nanoparticles that may not only enhance therapeutic activity but also mitigate concerns related to toxicity or skepticism surrounding phage use. This innovative approach not only addresses the challenges associated with phage therapy but also provides a platform for creating advanced drug delivery systems that mimic the natural processes observed in phage interactions. This strategy holds promise for improving the safety and acceptance of therapeutic interventions, offering a synthetic alternative that harnesses the benefits of phage therapy without its inherent limitations.

## Final remarks on how not to get lost in translation

Professor Lehr recommended harnessing delivery technologies developed in recent decades to address challenges related to potent drugs with poor bacterial bioavailability or high toxicity. This could be accomplished through local rather than systemic administration, for example, by developing aerosols for oral inhalation, mucoadhesive products for antibiotics, or smart delivery systems capable of accumulating in the target area and triggering drug release on site. On top of the technological aspects, also the economic aspects must be considered before pursuing this approach. When it comes to realizing emerging genome editing (i.e., CRISPR) or gene therapies in vivo, complex drug delivery technologies will be unavoidable. Without proper delivery, these therapies cannot reach their target and will fail to achieve the required therapeutic outcomes. Therefore, developing new drug delivery systems in the context of nucleic acid therapeutics seems to be inevitable.

Notably, Prestidge debates that the current traditional model in developing new novel therapies through classical medicinal chemistry is flawed and leads to higher failure rates down the pipeline because delivery solutions and drug delivery scientists are not engaged in product development in the early stages. The field is currently changing with industry, where drug delivery experts are increasingly being incorporated at all levels. Moreover, there is an increasing incorporation of artificial intelligence to design more fit-for-purpose drugs. Thus, the early involvement of formulation scientists can be a game changer.

In addition, Thomas points out that the key to facilitating major advances in the antimicrobial field is the willingness to take risks and invest in large-scale publicly funded programs to support novel solutions, where delivery technologies can be part of the answer. He also argues that there is a need to establish a framework from a legal and economic point of view to synergize the work done by academic institutions and the industry to incentivize collaborations and aid with product development. However, this shift in investors’ and policymakers’ funding priorities is only likely to stem from a shift in popular opinion in favor of awareness of AMR&T. Indeed, raising awareness of antibiotic use across multiple different industries is incredibly important. Various strategies may be suggested to do this. Roberts recommends two: better education of doctors who prescribe antibiotics, ensuring they are fully aware of the possible dangers of over-prescribing, and better education of the health authorities that are authorizing the over-the-counter use of these drugs. Roberts quotes, “We should not make it so easy for people to just buy any classic antibiotic, when we realize the trouble that they can cause.”

Moreover, Lehr strongly encourages all scientists to engage in science outreach programs to assist in the education of the public community about the appropriate use of antibiotics. For example, the HIPS at Saarland University holds different events on a yearly basis to bring awareness in society about different topics, including about different microbes found in the environment, and antibiotic use concerns. Prestidge acknowledges that increasing awareness needs to be a global approach. While developed countries have stewardship programs, developing countries are less likely to have the resources to do so. He explains that enormous investments are required from governments and health authorities in a global way to communicate how important this challenge is, and obviously, education is the key. In addition, Thomas adds that prevention is key and increasing awareness by communicating preventative strategies should be a priority.

Undoubtedly, drug delivery scientists hold a pivotal role in the battle against AMR&T. Drawing insights from the accounts of four esteemed experts within the field, a promising outlook for the future emerges. A robust understanding of the primary barriers impeding antimicrobial therapies has been established, laying the foundation for their improvement. In response, a plethora of effective tools and cutting-edge technologies are being developed to surmount these challenges. The critical phase involves translating these advancements into clinical applications and fostering awareness among prescribers, authorities, and communities regarding best practices for combating microbial infections and treatment. Throughout this process, delivery scientists have significant responsibility and are encouraged to step up, ensuring targeted and efficient delivery of antimicrobials. In conclusion, the dedication and innovation of drug delivery scientists are paramount in the ongoing fight against AMR, signifying their crucial contribution to safeguarding global health.

## Data Availability

No experimental data were used for this perspective article.

## Declarations

### Disclaimer

This perspective manuscript is part of thematic articles that belong to a collaboration between the Young Scientists Committee of the Controlled Release Society and Drug Delivery and Translational Research and is organized and edited by Dr. Tushar Kumeria and Dr. Hagar Labouta as DDTR Young Associate editors.
